# Posterior Reversible Encephalopathy Syndrome Associated with Tacrolimus in Cardiac Transplantation

**DOI:** 10.1155/2021/9998205

**Published:** 2021-06-24

**Authors:** Julián Alejandro Rivillas, Stephania Galindo-Coral, Francisco Arias-Mora, Juan David Lopez-Ponce de Leon, Noel Alberto Florez-Alarcón, Pastor Olaya-Rojas, Juan Esteban Gomez-Mesa

**Affiliations:** ^1^Resident of Neurology, Universidad Icesi, Cali, Colombia; ^2^Cardiology Department, Fundación Valle del Lili Hospital Universitario, Cali, Colombia; ^3^Clinical Research Center, Fundación Valle del Lili Hospital Universitario, Cali, Colombia; ^4^Neurology Department, Fundación Valle del Lili Hospital Universitario, Cali, Colombia

## Abstract

**Background:**

Neurological complications occur between 50 and 70% of patients with heart transplantation, including cerebrovascular events, infections, seizures, encephalopathy, and neurotoxicity due to pharmacological immunosuppression. Mortality associated with cerebrovascular complications is 7.5% in the first 30 days and up to 5.3% after the first month and up to the first year after transplantation. *Case Reports*. Three heart-transplanted patients (2 men and 1 woman) treated with tacrolimus were identified. They presented with posterior reversible encephalopathy syndrome on days 5, 6, and 58 posttransplantation, respectively. In these reported cases, no sequelae were observed at 6 months follow-up.

**Conclusions:**

Posterior reversible encephalopathy syndrome as a neurological complication in patients with HT occurred early after the procedure. Early diagnosis and treatment might reduce the risk of serious complications and mortality.

## 1. Background

Heart transplantation (HT) is a therapeutic option in patients with advanced and refractory heart failure. In Latin America, this procedure has been performed in more than 8,000 patients, in more than 85 institutions in 13 Latin American countries since 1968 [1, 2]. The current worldwide survival rate is up to 90% for the first year, with a median survival of 14 years. The patient's quality of life improves significantly, and up to 30% of them may reintegrate into working life [3]. After HT, different complications may occur, affecting the survival and functionality of patients, such as graft rejection, infections, neoplasms, high blood pressure, kidney disease, and neurological complications [4, 5].

Neurological complications occur between 50% and 70% of patients with HT when transient and mild effects are included. Most may occur within the first 30 days after the procedure and include cerebrovascular events, infections, seizures, encephalopathy, and neurotoxicity due to immunosuppressive medication. Neurological involvement is of particular concern because of its impact on patients' functional prognosis and survival [6, 7]. The most severe neurological complications are ischemic stroke followed by infection. Mortality associated with cerebrovascular complications is 7.5% in the first 30 days and up to 5.3% after the first month and up to the first year after transplantation. It can be as high as 20% in cases of opportunistic infections and cerebrovascular events [5, 8–10].

The most frequently maintained immunosuppressive agents used in heart transplants are corticosteroids, calcineurin inhibitors, and antimetabolites. Corticosteroids induce commonly acute neuropsychiatric symptoms including delirium, sleep disorders, depression, and cognitive decline. Cyclosporine and tacrolimus are both calcineurin inhibitors and can induce encephalopathy, seizures, akinetic mutism, and posterior reversible encephalopathy syndrome. Mycophenolate mofetil and azathioprine are antimetabolites. Mycophenolate mofetil does not have a significant direct neurotoxicity, but it may cause headaches on first doses. Azathioprine is rarely used for heart transplantation [6].

## 2. Clinical Case Reports

**Figure 1:**
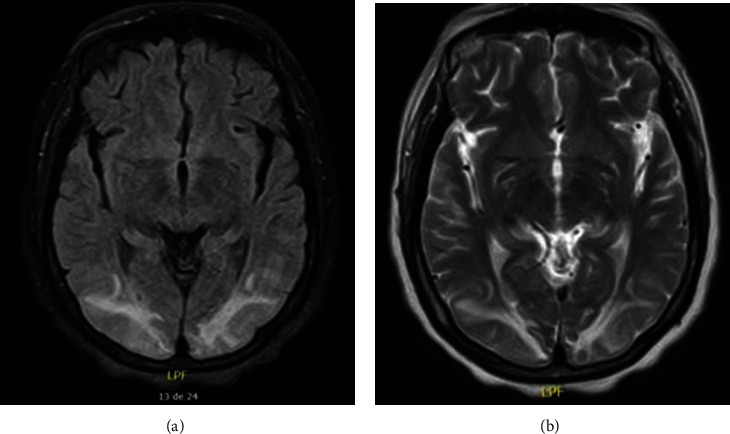
Brain MR of case 1. (a) Fluid-attenuated inversion recovery sequence showing occipital hyperintensities in occipital gyri. (b) T2-weighted sequence of the same patient showing hyperintensities in the occipital lobe.

**Figure 2:**
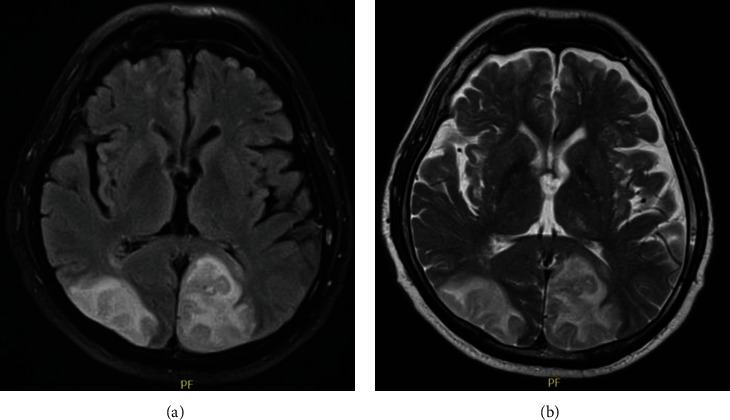
Brain MR of case 2. (a) Fluid-attenuated inversion recovery sequence showing extensive bilateral asymmetrical occipital hyperintensities in the occipital lobe and (b) its representation on T2-weighted sequence.

**Figure 3:**
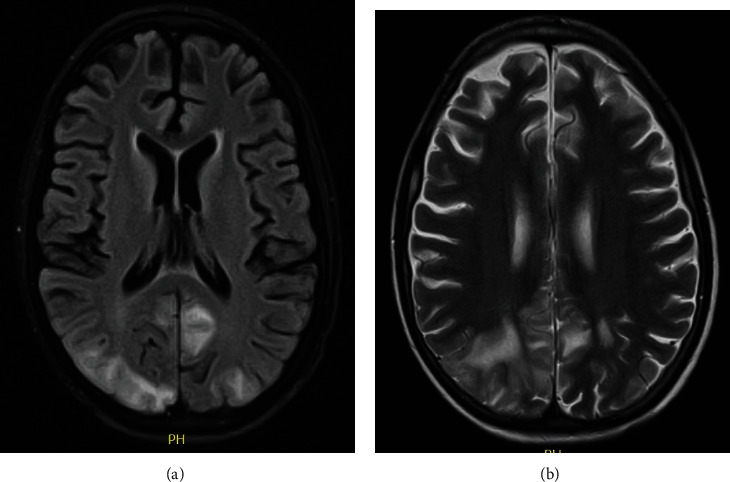
Brain MR of case 3. (a) Fluid-attenuated inversion recovery sequence showing occipitoparietal hyperintensities and (b) its representation in T2-weighted sequence.

Three cases of HT patients with PRES associated with the use of tacrolimus observed at different times after the procedure are described in a cohort of 223 heart transplant patients in a Colombian hospital. Clinical and radiological information is in Table 1 and Figures 1–3.

## 3. Discussion

PRES was initially described in 1996 [11] and has ever since been associated with clinical conditions such as high blood pressure, eclampsia, infections, autoimmune diseases, and the use of immunosuppressive drugs. The underlying pathophysiological mechanisms are not clear, but differences have been described in the vascular sympathetic innervation of the anterior and posterior brain circulation with a variable degree in the self-regulation capacity in the presence of external noxae as changes in cerebral perfusion pressure, microangiopathy, and toxic effects (i.e., tacrolimus). These tissue insults favor vasogenic edema in the parietal and occipital lobes occur [12]. In PRES associated with CI, vasoconstricting effects and direct endothelial damage have been described, which cause capillary leakage due to disruption of the blood-brain barrier, which ultimately causes cerebral vasogenic edema [13]. This effect is thought to be related to an intrinsic factor of inhibition of calcineurin widely expressed in the central nervous system. The absence of hypertension crisis and normal serum calcineurin values at diagnosis in reported cases in this article supports that hypothesis. The occurrence of PRES after heart transplantation is typically on the first 30 days after surgery with great variation among studies [14]. In published cases reviewed for this article, we found a median of 14 days after heart transplantation. Two of the cases presented here were below this mean time (Table 2). Since most of the reported cases are single-center case series, it is difficult to estimate the true incidence of the problem. Available data indicate that the incidence of PRES syndrome after solid organ transplantation was estimated to be between 1% and 6% [15], but more recent studies estimated it at 0.49% to 1.6% for heart transplants [16] At the end of 2020, our group had performed 223 heart transplants, so we calculate a cumulative incidence of 1.3 cases per 100 transplants since the start of the program in 1994.

### 3.1. Clinical Features, Imaging, and Histological Findings

PRES is characterized by an alteration of the level of consciousness, seizures, headache, visual disturbances, neurological deficit, and status epilepticus. Fugate and Rabinstein [17] proposed a 7-category list for patients with PRES: encephalopathy, seizure, headache, visual disturbance, focal neurological deficits, status epilepticus, and nausea/vomiting. All of the patients reported by us lacked the three latter. The typical triad of headache, visual disturbances, and seizures was present in all three reported cases. This triad seems to be present in 77% of cases according to previous studies. Other common symptom combination is seizures/encephalopathy (38%), seizures/headache (22.5%), and seizures/visual disturbances (14%) [17].

Brain resonance usually reports bilateral and asymmetric hyperintensities in T2-FLAIR sequences of the occipital and parietal regions, but transient lesions can also be found in the anterior region, cerebellum, centrum semiovale, pons, and hippocampus. Also, coexistent ischemic lesions can be present [18]. All reported cases showed typical brain MRI findings, but case 2 presented asymptomatic lesions in the frontal lobe. These findings may support a hypothesis of global alteration of the blood-brain barrier with posterior predominance. No other atypical or hemorrhagic lesions such as those described by McKinney et al. were found [19].

Histopathological findings of cellular edema, disruption of the blood-brain barrier, and, in some cases, necrosis are also observed [20]. These findings help to understand the pathophysiology of the disease but are rarely used in clinical practice. No histopathology information is available for our cases.

### 3.2. Risk Factors

The onset of symptoms usually occurs during the first month posttransplantation and rarely after the first year despite the chronic use of CI and dose titration during follow-up. The immediate postsurgical period and the following 30 days are critical, since the patient has been suffering from a chronic and debilitating disease with a significant compromise of the functional reserve and subsequently undergoes a major surgery with varying degrees of hemodynamic compromise, tissue hypoperfusion, and administration of immunosuppressive drugs. The reason for the greater incidence of PRES within the first 30 days is not known, but it may be favored by all the factors mentioned above [17]. Two of the reported cases developed PRES during the first 30 days and the other after 58 days, which was associated with a longer hospitalization time and evidence of graft dysfunction in the mediate the postoperative period. Other risk factors frequently reported as hemorrhage and diffusion restriction were not found in our patients [21].

### 3.3. PRES and Calcineurin Inhibitors

The harmful effects of CI on the endothelium do not seem to be dose-dependent, since many reported cases show normal serum tacrolimus levels at the time of PRES diagnosis (Table 1). That is the case of reported patients. They also lacked a history of hypertensive crises. Similar findings are reported by Song et al. [22]. In contrast, the systematic review performed by this same author reveals a history of hypertension in 69% of patients with PRES after transplantation. The reports of patients with cyclosporine-associated neurotoxicity included headache, seizures, and encephalopathy, and nausea is not necessarily associated with radiological findings of PRES [23, 24]. A trend toward an earlier occurrence and higher likelihood of PRES in the presence of normal drug levels in transplant recipients receiving cyclosporine than those receiving tacrolimus (median time to onset; 12 vs. 26 days and 40%, 8/20 vs. 25.8%, 8/31) [22].

### 3.4. PRES Treatment and Prognosis

Despite the lack of a general guideline, switching from tacrolimus to cyclosporine is a common measure in the cases published to date. Cyclosporine is effective in preventing organ rejection but has also a risk of causing PRES and other types of neurotoxicity [14]. However, given its high availability in developing countries, it is frequently used in cases of intolerance or adverse effect with other calcineurin inhibitors. Remarkably, in the six-month follow-up, our patients did not present relapse or any other type of neurotoxicity. It is feasible to think that the immunosuppressant is a factor in the early stages after surgery that contributes to the development of PRES but it is not the only cause. Implant rejections were not reported. Consequently, this strategy may seem to be safe and effective for the management of tacrolimus-associated PRES [25–27]. Other approaches include switching to sirolimus, everolimus, mycophenolate mofetil, or hydrocortisone and lowering the dose of immunosuppressive agent. Classically, a low incidence of sequelae, mortality, and recurrence has been described, as evidenced in these three cases. However, some reports report mortality and neurological sequelae as high as 15% especially when it is not identified and treated early and appropriately [28] (Table 2).

## 4. Conclusions

Neurological complications as PRES in HT patients can occur early after the procedure. Early diagnosis and treatment reduce the risk of more serious outcomes and associated mortality. CI-associated PRES in HT patients is a neurological complication that should be suspected when early “typical manifestations” of an altered level of consciousness, visual disturbances, and seizures appear. The timely diagnosis and treatment of events, change of immunosuppressive therapy to cyclosporine, and multidisciplinary follow-up could reduce the incidence of sequelae.

## Figures and Tables

**Table 1:** Description of patients with PRES after heart transplant.

	Case 1	Case 2	Case 3
Gender	Male	Male	Female
Age (years)	27	67	29
Etiology of HF	Becker muscular dystrophy	Ischemic heart disease	Chagas' heart disease
Onset of PRES symptoms (days)	6	5	58
Symptoms	Decreased visual acuity, generalized tonic-clonic seizures, altered level of consciousness	Blurred vision generalized tonic-clonic seizures, altered level of consciousness	Blurry vision generalized tonic-clonic seizures, altered level of consciousness, severe headache
Previous drugs	Tacrolimus MMF, methylprednisolone, TMP/SMX, acyclovir, vancomycin	Tacrolimus MMF, methylprednisolone, TMP/SMX, acyclovir, vancomycin, ceftriaxone	Tacrolimus MMF, prednisone, TMP/SMX, acyclovir, diltiazem, losartan
Hemoglobin (mg/dl)	9.0	10	8.5
Leukocytes (cel/ml)	6,470	8,540	14,130
Platelets (cel/ml)	130,000	140.000	441,000
Creatinine (mg/dl)	0,9	0,8	0,88
Sodium (mEq/l)	141	139	135
Potassium (mEq/l)	3.9	4.0	5.97
Glycemia (mg/dl)	138	165	145
Tacrolimus (ng/dl)	2.6	8.9	17.4
Brain MR	Bilateral and symmetrical parietal and occipital cortical-subcortical hyperintensities in the FLAIR and T2 sequences (Figure 1)	Asymmetric cortical hyperintensities in the parietal and occipital lobes (Figure 2)	Hyperintense lesions of posterior cortical and subcortical predominance without abnormal enhancements with the contrast agent (Figure 3)
Management	Discontinuing tacrolimus, starting cyclosporine, levetiracetam	Discontinuing tacrolimus, starting cyclosporine, levetiracetam	Discontinuing tacrolimus, starting cyclosporine, levetiracetam
Recurrence of symptoms at 6 months	None	None	None

HF: heart failure; PRES: posterior reversible encephalopathy syndrome; HT: heart transplantation; MMF: mycophenolate mofetil; TMP/SMX: trimethoprim-sulfamethoxazole; NMR: nuclear magnetic resonance.

**Table 2:** Reported cases compared with reported cases in Literature.

Ref	Sex	Age (years)	Days post-HT	Tacrolimus	Steroids	MMF	Sequelae
Reported cases							
—	M	27	6	Yes	Yes	Yes	No
—	M	67	5	Yes	Yes	Yes	No
—	F	29	58	Yes	Yes	Yes	No
Cases in literature							
1	F	68	14	Yes	No	Yes	No
1	M	19	44	Yes	No	Yes	No
2	F	26	7	Yes	Yes	Yes	No
2	M	33	15	Yes	Yes	Yes	No
3	M	52	8	Yes	Yes	Yes	Yes
4	M	37	16	Yes	Yes	Yes	No
5	F	32	5	Yes	Yes	Yes	No
6	F	30	45	No	Yes	Yes	No
7	F	22	5	Yes	No	Yes	No
8	M	10	10	Yes	Yes	Yes	No
9	F	54	14	Yes	No	Yes	No
10	M	18	150	Yes	Yes	Yes	No
11	F	20	60	Yes	Yes	Yes	No
12	M	10	4	Yes	Yes	Yes	No
13	M	12	4	Yes	Yes	Yes	No

Ref: reference; HT: heart transplantation; MMF: mycophenolate mofetil.

## Data Availability

No data were used to support this study.

## Abbreviations

CI: Calcineurin inhibitors
HF: Heart failure
HT: Heart transplantation
MMF: Mycophenolate mofetil
NMR: Nuclear magnetic resonance
PRES: Posterior reversible encephalopathy syndrome
TMP/SMX: Trimethoprim-sulfamethoxazol.
